# Effects of sleep deprivation on different phases of memory in the rat: dissociation between contextual and tone fear conditioning tasks

**DOI:** 10.3389/fnbeh.2014.00389

**Published:** 2014-11-07

**Authors:** Vanessa Contatto Rossi, Paula Ayako Tiba, Karin Di Monteiro Moreira, Tatiana Lima Ferreira, Maria Gabriela Menezes Oliveira, Deborah Suchecki

**Affiliations:** ^1^Department of Psychobiology, Escola Paulista de Medicina, Universidade Federal de São Paulo – UNIFESPSão Paulo, Brazil; ^2^Centro de Matemática, Computação de Cognição, Universidade Federal do ABC – UFABCSanto André, Brazil

**Keywords:** learning, memory, sleep deprivation, acquisition, consolidation, retrieval, extinction

## Abstract

Numerous studies show that sleep deprivation (SD) impacts negatively on cognitive processes, including learning and memory. Memory formation encompasses distinct phases of which acquisition, consolidation and retrieval are better known. Previous studies with pre-training SD induced by the platform method have shown impairment in fear conditioning tasks. Nonetheless, pre-training manipulations do not allow the distinction between effects on acquisition and/or consolidation, interfering, ultimately, on recall of/performance in the task. In the present study, animals were first trained in contextual and tone fear conditioning (TFC) tasks and then submitted to SD with the purpose to evaluate the effect of this manipulation on different stages of the learning process, e.g., in the uptake of (new) information during learning, its encoding and stabilization, and the recall of stored memories. Besides, we also investigated the effect of SD in the extinction of fear memory and a possible state-dependent learning induced by this manipulation. For each task (contextual or TFC), animals were trained and then distributed into control, not sleep-deprived (CTL) and SD groups, the latter being submitted to the modified multiple platform paradigm for 96 h. Subsets of eight rats in each group/experiment were submitted to the test of the tasks, either immediately or at different time intervals after SD. The results indicated that (a) pre- but not post-training SD impaired recall in the contextual and TFC; (b) this impairment was not state-dependent; and (c) in the contextual fear conditioning (CFC), pre-test SD prevented extinction of the learned task. Overall, these results suggest that SD interferes with acquisition, recall and extinction, but not necessarily with consolidation of emotional memory.

## Introduction

A reciprocal influence between sleep and memory has been suggested, insofar as consolidation of newly acquired information is facilitated by previous sleep periods, whereas acquisition of new information alters sleep pattern (Maquet, 2001; Stickgold et al., 2001; Smith et al., 2004; Walker and Stickgold, 2004, 2005). Additional evidence of this relationship has been given by human and animal studies demonstrating that training in different memory tasks increases sleep time (Lucero, 1970; Smith et al., 1980; Smith and Lapp, 1986; Portell-Cortés et al., 1989; Smith and Rose, 1997) and that either sleep- (SD) or REM sleep deprivation (REMSD) before training impairs the performance of animals in numerous hippocampal-dependent tasks, such as inhibitory avoidance (Stern, 1971; Bueno et al., 1994, 2000; Gruart-Masso et al., 1995; Moreira et al., 2003; Dubiela et al., 2005), multiple trial inhibitory avoidance (Moreira et al., 2010; Ota et al., 2013), Morris water maze (Smith and Rose, 1996; Guan et al., 2004) and fear conditioning (Hicks et al., 1988; Dametto et al., 2002; McDermott et al., 2003; Tiba et al., 2008). However, results from studies using pre-training protracted REMSD protocols are difficult to interpret because the animals are under an altered sleep-waking pattern both before (sleep-deprived) and after training (when sleep rebound is taking place) thus, precluding conclusions as to whether this manipulation affects acquisition and/or consolidation of the information in these memory tasks. In addition, REMSD-induced memory impairment may be due to its effect on attention, since this is disrupted by 72 or 96 h of REMSD and recovered by 24 h of sleep rebound (Godoi et al., 2005), suggesting that acquisition may be compromised. Indeed, sleep-deprived rats require more trainings in the multiple inhibitory avoidance task than control rats, and even so, their performance is poorer when tested 24 h later (Moreira et al., 2010; Ota et al., 2013). Finally, the possibility exists that impairment of performance in the abovementioned studies is due to a phenomenon known as state-dependent learning. This phenomenon implies in learning a particular response under a given stimulus-situation that involves a conditioning aspect; in test situations, when the training stimulus is replaced by another one, there is no transfer of the learned response (Overton, 1974). Therefore, in state-dependent learning a particular response is displayed if, and only if, there exists a similarity of the animal’s inner state between the training and testing conditions (Colpaert et al., 1976).

Studies employing passive avoidance task show that 24 h of sleep recovery are sufficient to restore the performance of 96 h REM sleep-deprived rats (Dubiela et al., 2005, 2010), suggesting that SD-induced performance deficit may not be due to impairment of consolidation, but rather to some other unspecific effect, such as altered motor performance. In fact, REMSD induced-increased locomotor activity in rats has been reported by some authors in rats (Suchecki et al., 2002; Tartar et al., 2009) and mice (Armani et al., 2012), but not by others (Dubiela et al., 2011); in the case of passive avoidance task, altered motor activity could be responsible for the short- (2 h post-training), but not for the long-term (24 h post-training) retention deficit (Dubiela et al., 2005), indicating, once again, that acquisition is impaired by REMSD.

One strategy to circumvent the impact of SD on acquisition is to sleep-deprive animals after training, so acquisition is guaranteed. The studies that employed this approach resulted in controversial results, with impairing (Graves et al., 2003; Palchykova et al., 2006), as well as improving effects (Gisquet-Verrier and Smith, 1989; Smith, 1996; Smith et al., 1998). This discrepancy may be attributed to the length (5 h/6 h × 24 h) and to the type of SD (total × REMSD). Moreover, the interval between SD and testing is an important methodological issue that should be considered when evaluating its effects in different memory stages. For instance, Silva et al. (2004) showed that 72 h of REMSD after training did not induce memory impairment in passive avoidance test or in the plus maze discriminative test when testing took place immediately after the end of SD, but when animals were re-tested 1 week later, they displayed impaired performance. Whether this impairment represents a facilitated extinction of the fear memory was not explored in this study, but there is evidence that 6 h of REMSD retard extinction of cued fear memory (Silvestri and Root, 2008).

Given the controversy regarding the effects of protracted SD on mnemonic process, the aim of the present study was to investigate the consequences of this manipulation, applied in different moments of memory formation in two related tasks that recruit distinct neural circuits, contextual fear conditioning (CFC), a hippocampal-dependent task, and tone fear conditioning (TFC), a hippocampal-independent task. Moreover, we also tested whether the performance impairment observed in these tasks could be explained by the state-dependent learning.

## Methods

### Animals

Male Wistar rats, aged 3 months, were obtained from the breeding colony of the Department of Psychobiology–UNIFESP. The animals were kept in groups of four in plastic cages (40 × 30 × 17 cm), filled with sawdust bedding, in a room under controlled temperature (23°C ± 2°C) and light/ dark cycle (lights on from 07:00 h to 19:00 h), with food and water provided ad *libitum*. The experimental protocols were approved by the Animal Care and Use Committee of UNIFESP (CEP # 0943/06) and were in accordance with National Institutes of Health guidelines on animal care.

### SD procedure

Rats were sleep deprived by the modified multiple platform method for 96 h. SD was conducted by placing eight rats in a large water tank (145 × 30 × 41 cm) containing 12 narrow platforms (6.5 cm in diameter). Previous studies from our group show that the platform method (either single or multiple) completely abolishes REM sleep but also decreases slow-wave sleep by approximately 35–40% (Machado et al., 2004, 2008, 2013). The presence of cage-mates and multiple platforms prevent social isolation and movement restriction associated with earlier versions of SD (Suchecki and Tufik, 2000). Animals in the control (CTL) group remained in their home cages (HCs) in the same room where the SD procedure took place and were placed daily in the water tank for 1 h, between 12:00 h and 13:00 h. This procedure was implemented so both sleep deprived and CTL groups would be removed from the same environment (water tank) before being submitted to the different protocols.

### Apparatus

The fear conditioning task apparatus consisted of an acrylic box, measuring 21 × 26 × 27.5 cm. The apparatus had black walls and transparent acrylic top. The floor consisted of a metal grid (0.4 cm diameter rods placed 1.2 cm apart) connected to a shock generator and control module (AVS—Projetos Especiais, São Paulo, Brazil), through which footshocks could be delivered.

The TFC test was carried out using a white cylindrical test chamber (35 cm in diameter × 60 cm high), with a transparent lid containing small holes. The apparatuses were placed in different rooms, being connected to a buzzer located outside the conditioning apparatus and outside the cylindrical chamber (60 dB) activated by a manual switch and used as the conditioned stimulus (CS).

### Behavioral procedure

*Contextual fear conditioning (CFC)*: Training was performed in one session, in which the animals were individually placed in the conditioning chamber where they remained for 2 min. The behavior of each animal was recorded continuously by measuring the time in freezing behavior (defined as complete immobility and absence of vibrissae movements) minute by minute. After this period, rats received five footshocks (0.7 mA, 1 s long) at 30 s intervals and were removed from the apparatus 1 min after the last footshock. CFC tests were performed after different post-training periods, depending on the experiment protocol. During the test, rats were placed in the same training context, and no footshock was delivered. The time in freezing was again recorded minute by minute for 5 min. Freezing time/min ratio was taken as the performance of contextual conditioning.

*Tone fear conditioning*: Training was performed in one session, identical to CFC, but in this case, a sound stimulus (60 dB–5 s long) was delivered, finishing concomitantly with a 1 s long footshock (0.7 mA). Five tone-footshock pairings were delivered, 30 s apart. During the TFC test (which also took place after different time intervals after training), rats were placed in the cylindrical chamber (new context, different room) and after 2 min the sound was presented 5 times, in the same schedule as during training, without release of any footshock and the freezing time /min was taken as the conditioning measure.

### Experiments 1, 2 and 3

**Figure 1:**
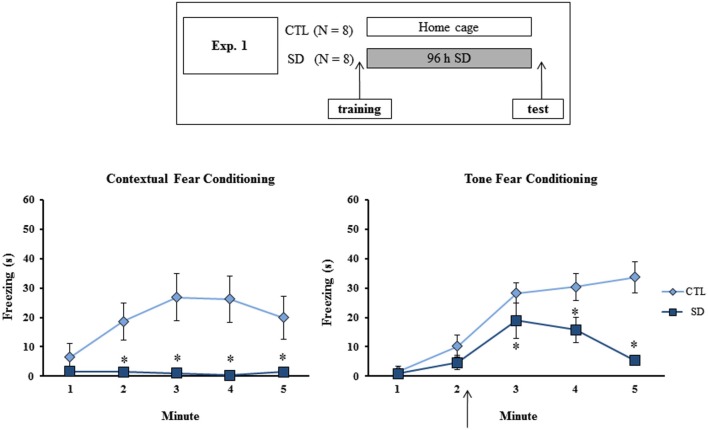
**Experimental procedure and results from Experiment 1**. Freezing time (s) expressed during contextual (left panel) and tone (right panel) fear conditioning tests for sleep-deprived and control animals (CTL). The arrow indicates the moment of first tone presentation (after minute 2 in TFC). Sleep deprivation was applied immediately in between training and test sessions. SD—Sleep deprivation; HC—home cage; values are expressed in mean ± S.E.M. of eight animals/group/task. * —different from CTL group (two-way ANOVA, Group × Minute interaction).

**Figure 2:**
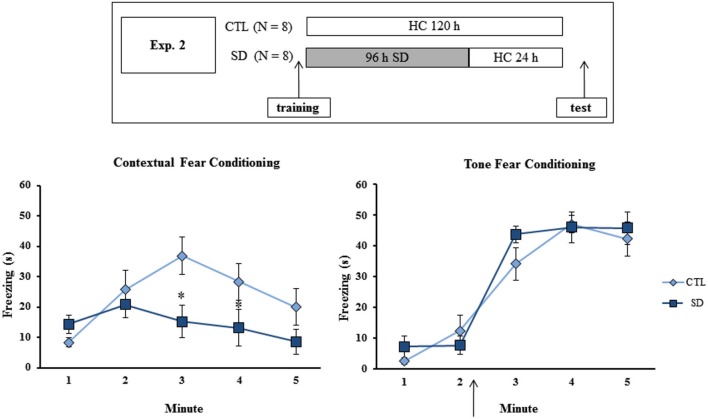
**Experimental procedure and results from Experiment 2**. Freezing time (s) expressed during contextual (left panel) and tone (right panel) fear conditioning tests for sleep-deprived and control animals (CTL). The arrow indicates the moment of first tone presentation (after minute 2 in TFC). Sleep deprivation was applied after training session and test session occurred after 24 h in which the animals remained in their home cages. SD—Sleep deprivation; HC—home cage; values are expressed in mean ± S.E.M. of eight animals/group/task. * —different from CTL group (two-way ANOVA, Group × Minute interaction).

**Figure 3:**
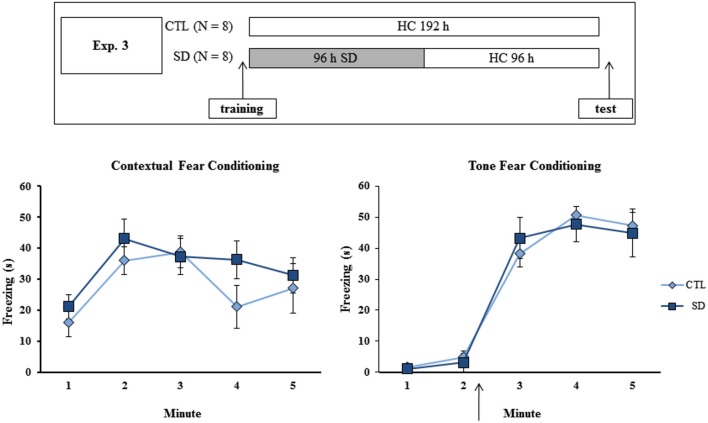
**Experimental procedure and results from Experiment 3**. Freezing time (s) expressed during contextual (left panel) and tone (right panel) fear conditioning tests for sleep-deprived and control animals (CTL). The arrow indicates the moment of first tone presentation (after minute 2 in TFC). Sleep deprivation was applied after training session and test session occurred after 96 h in which the animals remained in their home cages. SD—sleep deprivation; HC—home cage; values are expressed in mean ± S.E.M. of eight animals/group/task. * —different from CTL group (two-way ANOVA, Group × Minute interaction).

Different groups of animals (*N* = 8/group/task/experiment) were trained in the CFC or in the TFC and then distributed into either 96 h of SD or CTL groups. In Experiment 1A, the groups were tested in the respective tasks immediately after the end of the SD period (or at the equivalent time for the CTL group) (Figure 1, upper panel). In Experiment 2, rats in the SD group were allowed to sleep for 24 h before the test, in order to examine whether the manipulation interfered with memory consolidation or retrieval (Figure 2, upper panel). In Experiment 3, sleep-deprived animals were allowed to sleep for 96 h before being tested, a period sufficient to guarantee return to normal sleep pattern (Machado et al., 2004). The experimental procedure is shown in Figure 3 (upper panel).

### Experiment 4

**Figure 4:**
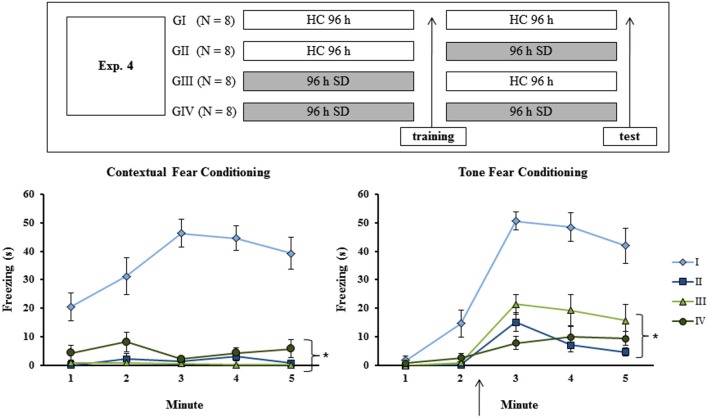
**Experimental procedure and results from Experiment 4**. Freezing time (s) expressed during contextual (left panel) and tone (right panel) fear conditioning test for sleep-deprived and control animals (CTL). The arrow indicates the moment of first tone presentation (after minute 2 in TFC). Sleep deprivation was applied at different moments for each group. To confirm state dependent learning, groups that were in the same state during both training and test session (G I and G IV) should perform better than the others (G II and G III). SD—sleep deprivation; HC—home cage; values are expressed in mean ±S.E.M. of eight animals/group/task. * —all groups were different from G I (CTL group —two-way ANOVA, Group effect).

To discard the possibility that the results of Experiments 1A, B and C were due to a state-dependent learning, different sets of animals were distributed in one of four groups: G I–remained in the HC for the whole experimental period; G II–Submitted to SD only after training; G III–submitted to SD only before training; G IV–submitted to SD both before and after training, thus being sleep-deprived during acquisition and retrieval of the learned material. Consequently, both G I and G IV were in the same state during both training and test sessions, and if indeed there was a state-dependent learning then these groups should not exhibit memory impairment. On the other hand, if memory impairment were observed only for pre-training or pre-test sessions as shown previously (both in the present study and in the literature), then only G I should perform adequately during the test (the experimental procedure is shown in Figure 4, upper panel).

### Experiment 5

**Figure 5:**
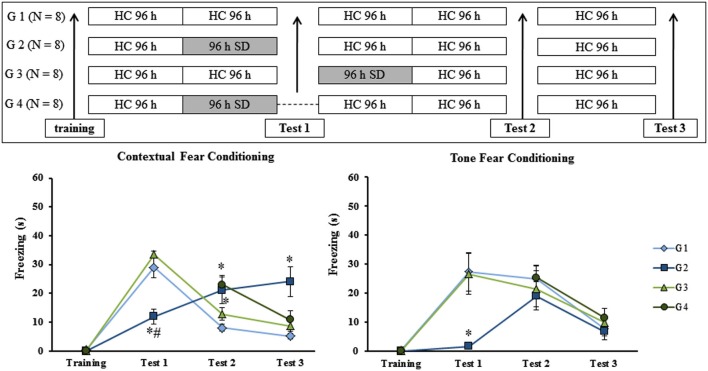
**Experimental procedure and results from Experiment 5**. Freezing time (s) expressed during contextual (left panel) and tone (right panel) fear conditioning test for sleep-deprived and control animals (CTL). Sleep deprivation was applied at different moments for each group: before test 1 for G2; after test 2 for G3 and for G4, sleep deprived animals were not submitted to test 1. SD—sleep deprivation; HC—home cage; values are expressed in mean ±S.E.M. of eight animals/group. * —different from G 1 (CTL group—one-way ANOVA, Group effect).

The previous results suggested that SD impaired recall of context fear conditioning. However, it is not possible to completely rule out the possibility that SD impaired the ability of animals to freeze (an effect on motor activity). To rule out this possibility, we exposed trained rats to three tests in CFC and TFC, separated by 8 days from each other. Different groups of animals were trained and then submitted to 96 h of SD at different moments: before test 1 (G2), after test 1 (G3) or in the absence of the first re-exposure to the conditioning chamber (G4). CTL group (G1) remained in the HC during the entire experimental protocol as can be seen in Figure 5 (upper panel).

### Statistical analysis

Data from Experiments 1A, B and C, were analyzed by a two-way ANOVA for repeated measures, with Group (SD × CTL) and Minute (repeated measure: 1–5) as main factors. For Experiment 2, a two-way ANOVA for dependent variables was employed with Group (G I–G IV) and Minute (repeated measure: 1–5) as main factors. Analysis of Experiment 3 used a one-way ANOVA for each session (training, test 1, test 2 and test 3), considering four groups (G1–G4; except for test 1, in which G4 was not tested). When necessary, a *post hoc* analysis was performed by the Newman-Keuls test, with a *P*-value ≤ 0.05 being considered statistically significant.

## Results

### Experiment 1: Effects of SD immediately before testing in fear conditioning tasks

During training session, no difference in freezing time between the groups was observed on either task (data not shown). During CFC test, main effects of Group (*F*_(1,14)_ = 9.66; *p* < 0.01) and Minute (*F*_(4,56)_ = 3.82; *p* < 0.01) and an interaction between these factors (*F*_(4,56)_ = 4.67; *p* < 0.005) were revealed, where SD group exhibited less freezing time than CTL animals at 2, 3, 4 and 5 min (Figure 1, left panel).

In the TFC task, main effects of Group (*F*_(1,14)_ = 9.37; *p* < 0.01) and Minute (*F*_(4,56)_ = 24.23; *p* < 0.0001) and an interaction between these factors (*F*_(4,56)_ = 6.65; *p* < 0.001) were also observed. *Post-hoc* analysis showed that SD rats displayed less freezing time than CTL rats after the tone, although the groups did not differ before presentation of the CS (Figure 1, right panel).

### Experiment 2: Effects of 24 h sleep recovery on testing of fear conditioning tasks

No difference in freezing time was found during training of either task, or for the test in TFC. However a Group × Minute interaction was found (*F*_(4,52)_ = 4.56; *p* < 0.003) for CFC and the *post hoc* analysis revealed that SD animals displayed less freezing during minutes 3 and 4 than CTL rats (Figure 2).

### Experiment 3: Effects of 96 h sleep recovery on testing of fear conditioning tasks

No difference in freezing time was found during training or test of either task. In TFC, a Minute effect was found (*F*_(4,52)_ = 98.75; *p* < 0.001), and freezing behavior was equally increased after tone presentation in both CTL and SD groups (Figure 3, left panel).

### Experiment 4: State-dependent effect of SD on fear conditioning tasks

Analysis of CFC revealed a Group effect for training session (*F*_(3,28)_ = 7.84; *p* < 0.0001), as well as a Minute effect (*F*_(2,56)_ = 136.02; *p* < 0.001) and a Group × Minute interaction (*F*_(6,56)_ = 11.96; *p* < 0.001). The Newman-Keuls test showed that freezing time was lower for GI (CTL group) and GII (rats sleep-deprived immediately before testing) than for GIII (rats sleep-deprived immediately before training) and GIV (rats sleep-deprived immediately before training and before testing), which were not different from each other (data not shown). Analysis of the animals’ performance during the test revealed main effects of Group (*F*_(3,28)_ = 51.64; *p* < 0.001) and Minute (*F*_(4,112)_ = 6,79; *p* < 0,0001) and an interaction between these factors (*F*_(12,112)_ = 7.52; *p* < 0.001). Newman-Keuls test showed that GI was different from all other groups, which did not differ from each other (Figure 4, left panel).

For TFC training, there was no Group effect or Group × Minute interaction, but only a Minute effect (*F*_(2,48)_ = 89.69; *p* < 0.001). During test, two-way ANOVA revealed main effects of Group (*F*_(3,24)_ = 35.21; *p* < 0.001) and Minute (*F*_(4,96)_ = 42.96; *p* < 0.0001) and a Group × Minute interaction (*F*_(12,96)_ = 7.03; *p* < 0.001); GI was different from all other groups, which were not different from each other (Figure 4, right panel).

### Experiment 5: Effect of SD on multiple tests in fear conditioning tasks

No differences were observed during training sessions in either task. In regard to the animals’ performance in the CFC, there was a main effect of Group on test 1 (*F*_(2,20)_ = 20.66; *p* < 0.00005), test 2 (*F*_(3,27)_ = 4.76; *p* < 0.01) and test 3 (*F*_(3,27)_ = 6.32; *p* < 0.005). *Post hoc* analysis showed that on test 1, G2 was different from G1 (CTL group) and G3 (rats sleep-deprived immediately before test 1; *p* < 0.0005); on test 2, G2 and G4 (rats were sleep-deprived as G2, but were submitted to test 1) were different from G1, but not from G3 and on test 3, again, only G2 was different from all other groups (*p* < 0.01) (Figure 5, left panel).

For TFC, there was no difference among groups during training, test 2 or test 3. For test 1, a Group effect was found (*F*_(2,21)_ = 7.90; *p* < 0.005) and the *post hoc* analysis revealed that only G2 was different from G1 and G3 (*p* < 0.01; Figure 5, right panel).

## Discussion

The results of the present study showed that SD affected memory acquisition and retrieval, but not consolidation; this effect was not induced by a state-dependent learning and exposure of animals to SD only immediately before the first retrieval session impaired the extinction of a hippocampus-dependent task.

In Experiments 1, 2 and 3, we investigated whether SD-induced impairment of recall in aversively motivated tasks, in the present case, CFC and TFC, resulted from interference with memory consolidation and/or recall *per se*. The results of these experiments strongly suggest that SD impairs recall, but not consolidation, because the performance of sleep-deprived rats in the TFC task was restored to control values after 24 h of sleep recovery, whereas in the CFC, the same effect was obtained between 24 and 96 h of sleep recovery. Interestingly, previous studies have shown that short periods of SD immediately (Graves et al., 2003; Palchykova et al., 2006) or 1 h (Prince et al., 2014) after training in hippocampus-dependent tasks impair memory consolidation and, consequently, recall. One possible explanation for the lack of SD effect on consolidation, may reside in the amount of associations made between footshock and either context or tone; in Graves et al. (2003), mice received one footshock of 1.5 mA, whereas in the present one, rats received five footshocks of 0.8 mA, and there is a possibility that memory consolidation begins already after the first pairing. Nonetheless, this possibility appears to be true for CFC, whereas data on the effects of SD on TFC are controversial, with studies showing this task to be resistant to the disrupting effects of REMSD (Bueno et al., 1994; Ruskin et al., 2004; Ruskin and Lahoste, 2009) or SD (Graves et al., 2003) and others showing that it is also affected by SD (Dametto et al., 2002; Kumar and Jha, 2012). These findings may explain why a shorter period of sleep recovery was already enough to restore the animals’ performance in TFC, whereas in CFC, a period between 24 h and 96 h was required for animals to express their recall-related behavior.

It has been shown previously that 96 h of SD by the platform method leads to intense sleep rebound in the 24 h following the deprivation period (Machado et al., 2004, 2008, 2013) resulting in disorganized sleep pattern characterized by excessive compensation of REM sleep and reduced non-REM sleep. Impairment of recall could also be attributed to this altered sleep architecture as recent studies have shown that non-REM sleep is also important for memory performance in emotional tasks (Giuditta et al., 1995; Fogel and Smith, 2006; Hellman and Abel, 2007). By allowing animals to sleep for 96 h between SD and testing, sleep pattern as well as the performance of sleep-deprived rats returned to normal on both tasks, being identical to that of control rats, thus evidencing that SD affects retrieval, but not memory consolidation. In addition, these data indicate a dissociative effect of SD on CFC and TFC, reinforcing the proposition that the impact of SD is not unspecific (as, for instance, on motor activity) since both tasks require the same response: freezing behavior.

The results of Experiment 4 indicated that memory impairment in both CFC and TFC was not due to a state-dependent learning. If it were, then animals that were sleep deprived before training and before testing should display a performance similar to that of control animals. However, the performance in both tasks was impaired in animals sleep-deprived either before training and/or testing. These results are in contrast with a previous one (Patti et al., 2010), that investigated the state-dependent effects of 72 h of REMSD in mice. Possible sources of controversy may be the use of different species, length of SD, but most of all, differences in the experimental protocol, for in Patti et al.’s study, testing was performed 7 days after training, a time sufficient for the animals to sleep recover and, according to the present results, to restore the performance in hippocampus-dependent tasks. In the present study, there was no interval between training and testing other than the period involving SD.

Just as REMSD applied before training may impair performance by altering attention state during acquisition so can the same phenomenon be true for retrieval. As, to the best of our knowledge, no study had ever been performed to evaluate the effects of SD on attentional process during retrieval, this possibility cannot be ruled out. Thus, even if acquisition and consolidation phases were guaranteed (Experiments 1B and C), pre-test SD might still affect unspecific aspects of performance rather than impairing retrieval *per se*. Importantly, during retrieval, memories become labile and may undergo two different processes: one that weakens old memories as a result of extinction and another that strengthens the memory as a result of additional consolidation, a process known as reconsolidation. These processes share common mechanisms but also recruit distinct circuits (for review, see (Alberini, 2005). Carrying out subsequent tests, in which the animals would be no longer sleep-deprived, could be a useful strategy to assure that normal performance was restored and to evaluate specific aspects of memory retrieval. Thus, in Experiment 5, animals were sleep-deprived either immediately before or after the first test, in order to investigate whether the manipulation would interfere with post-learning mechanisms. The results confirmed the impairing effects of SD before testing, but fear memory was expressed again in a subsequent test of both CFC and TFC, when rats had already recovered from SD (test 2). As expected, all groups, except from that sleep-deprived before test 1 of CFC exhibited low levels of freezing behavior upon repeated exposures to the CS, indicating that SD before recall of hippocampus-dependent fear memory had a long-term effect on memory processing. This result is unlikely to reflect SD-induced fear memory erasure, since SD when not followed by testing (G4 in Experiment 5) did not affect memory extinction. Interestingly, although freezing behavior was not expressed by rats of G2, their memory became labile, allowing some kind of alteration to take place leading to persistence of the fear memory. Several possibilities can be offered to explain the effect of SD conducted just prior to the first test. The first one refers to the acute effect on memory retrieval: although this may be the case, it is not possible to dissociate this effect from the confounding effect of SD on motor performance or emotionality. Usually a retrieval deficit can be inferred when the manipulation affects only one task, but in the present experiments both tasks were affected. Nonetheless, the long lasting effect of SD selectively observed in CFC cannot be attributed to an acute influence on recall or motor performance. The second one refers to the impairing effect of SD on extinction or, alternatively, on reconsolidation, or both. As control rats extinguished the conditioned response it is reasonable to assume that sleep manipulation interfered with this phenomenon. Because stressful situations disrupt reconsolidation (Cai et al., 2006) and SD induces sustained increase of corticosterone plasma levels (Galvão et al., 2009), it is unlikely that SD would produce a positive effect on reconsolidation of fear memories.

Pre-test SD affected memory extinction only for CFC, but not for TFC task, a result that is in contrast with previous work reporting that REMSD affects only TFC but not CFC (Silvestri, 2005; Fu et al., 2007). Some methodological differences may explain this contradiction. In Silvestri’s work, rats were submitted to 6 h of REMSD immediately after training, whereas testing/extinction training occurred 48 h later. Sleep-deprived animals did not extinguish fear behavior in TFC, but freezing was reduced in CFC. In a subsequent test, no difference was found in TFC, and all animals expressed similar levels of fear behavior, whereas there was a tendency to increase freezing in REMSD animals in CFC. This later result would agree with the present data, in which SD animals presented impaired extinction in subsequent tests only in CFC. Importantly, in Silvestri’s work training/testing and extinction for TFC were carried out in the same chamber, while in the present study TFC test and extinction was performed in a chamber different from the training one. The importance of context on modulation of extinction performance has been reviewed elsewhere (Bouton et al., 2006). Although Fu et al.’s work took this aspect into consideration, the same result was reported, i.e., impaired extinction in the TFC but not in the CFC after SD. There are still some methodological differences among these studies that could account for the distinct outcomes reported, including habituation prior to training (used in both studies–20 and 30 min respectively—but not in the present one), that could facilitate contextual extinction, masking possible effects of REMSD on extinction of this task, different lengths of SD (6 h after training in both studies), interval between training and testing (shorter periods in the previous studies and 96 h in the present one) and different methods of SD. Naturally, it is only possible to establish which factor(s) could influence the outcomes with certainty, by performing a study that compares all these variables.

Nevertheless, despite the inconsistencies between the abovementioned studies and the present one, the outcomes seem to convey the message that sleep loss can be detrimental for extinguishing aversive memories. This conclusion is in accordance with reports in human beings who had REMS periods during a 90-min nap and displayed less reaction to fearful facial expressions and more positive reactions to happy faces (Gujar et al., 2011) and a report that individuals who were involved in automobile accidents were more likely to develop depression and posttraumatic stress disorder (PTSD) when experienced sleep disturbances on the 2 weeks prior to the traumatic event (Bryant et al., 2010); conversely, individuals who displayed consolidated REM sleep in the aftermath of a traumatic situation are less likely to develop PTSD (Mellman et al., 2007). Collectively, these findings agrees with the proposition that REM sleep facilitates reframing of negative experiences and restores emotional reactivity (Walker and Van Der Helm, 2009). Therefore, experimental protocols such as those used in Silvestri (2005), Fu et al. (2007) and in Experiment 3 are promising tools to study the neural circuits involved in the relationship between REM sleep and extinction of fear memory.

In conclusion, 96 h of SD immediately after training of aversive memory tasks impaired the recall of fear memory both in context and TFC and this effect was reversed after a period of sleep recovery, but did not seem to result from a state-dependent learning process. However, when SD took place immediately before testing, there was an impairment of extinction of the aversive memory only in CFC.

## Conflict of interest statement

The authors declare that the research was conducted in the absence of any commercial or financial relationships that could be construed as a potential conflict of interest.
